# The association between dietary glycemic and insulin indices with incidence of cardiovascular disease: Tehran lipid and glucose study

**DOI:** 10.1186/s12889-020-09586-5

**Published:** 2020-10-02

**Authors:** Farshad Teymoori, Hossein Farhadnejad, Parvin Mirmiran, Milad Nazarzadeh, Fereidoun Azizi

**Affiliations:** 1 Nutrition and Endocrine Research Center, Research Institute for Endocrine Sciences, Shahid Beheshti University of Medical Sciences, P.O. Box: 19395-4741, Tehran, Iran; 2 Department of Nutrition, School of Public Health, Iran University of Medical Sciences, Tehran, Iran; 3 The George Institute for Global Health, University of Oxford, Oxford, UK; 4 Endocrine Research Center, Research Institute for Endocrine Sciences, Shahid Beheshti University of Medical Sciences, Tehran, Iran

**Keywords:** Insulin index, Insulin load, Glycemic index, Glycemic load, Cardiovascular disease, CVD, Adult

## Abstract

**Background:**

The present study was conducted to investigate the association of dietary insulin index(II), insulin load(IL), glycemic index(GI), and glycemic load(GL) with the risk of cardiovascular disease(CVD).

**Methods:**

This cohort study was conducted within the framework of the Tehran Lipid and Glucose Study on 2198 subjects, aged≥19 years old, who were followed-up for a median (IQR) 6.7 (6.1–7.1) years. Dietary GI, GL, II, and IL were calculated using a food frequency questionnaire at the baseline. Multivariate Cox proportional hazard regression models were used to estimate the risk of CVD across quartiles of dietary insulin and glycemic indices.

**Results:**

Mean ± SD age of the subjects(44.9% men) was 38.3 ± 13.4 years. During a mean of 2406 ± 417 person-years of follow-up, 76(3.5%) new cases of the CVD were ascertained. The mean ± SD of II, IL, GI, and GL of participants were 51.7 ± 6.5, 235.8 ± 90.2, 61.9 ± 7.8, and 202.2 ± 78.1, respectively. After adjusting for the variables of age, sex, smoking, physical activity, daily energy intake, body mass index, diabetes, and hypertension, the hazard ratio (HR) of the highest quartile of dietary GL was 2.77(95%CI:1.00–7.69,P for trend:0.033) compared to the lowest one. Also, each one SD increase in the GL score was associated with a higher risk of CVD[(RR:1.46;CI:1.00–2.16),*P*-value = 0.047]. However, there was no significant association between the dietary GI, II, and IL and risk for CVD incidence.

**Conclusions:**

Our results suggested that a high GL diet can increase the incidence of CVD, whereas high dietary II and IL were not associated with the risk of CVD among adults.

## Background

Cardiovascular disease (CVD), as the main global health problem causes a greater rate of comorbidities and mortality worldwide [1]. The obesity, elevated blood pressure, dyslipidemia, hyperglycemia, and insulin resistance (IR) are the main risk factors of CVD [2, 3], all of which are influenced by the dietary intakes. Several studies have shown the associations between the low fiber diet and overweight and IR, high sodium intake and hypertension, high saturated fatty acids and hypercholesterolemia and atherosclerosis, as well as a diet rich in the refined grain and simple sugar and hyperglycemia and IR [4–6].

Glycemic index (GI) and glycemic load (GL) are commonly used for the estimation of the insulinogenic effects of the foods and indicate the rate of the increase in the blood sugar after consumption of the carbohydrate-containing foods [ 7]. However, the serum response of glucose is not necessarily in accordance with the insulin response. Although, the insulin response is almost induced by the carbohydrates, other nutrients such as dietary protein and fats can influence the insulin secretion [8]. So, based on the concept of insulinogenic effects of the foods on the development of the chronic disease, recently, insulin indices (IIs) of the foods including dietary insulin index (II) and insulin load (IL) have been introduced, which can predict the overall insulin load of the main part of the diet; consequently estimating the odds for the occurrence of hyperinsulinemia [8, 9]. As the IIs compute based on the calorie content of the foods, rather than the glycemic indices, determined based on the carbohydrate content of the foods, they also provide the ability for assessing the insulin response induced by low or free-carbohydrate foods.

Previous reports indicate a direct association between the GI, and GL and the CVD risk factors such as high low-density lipoprotein -cholesterol and diabetes; however, findings about the incidence of CVD are not definite, and they seem to be a risk factor of CVD among the women, not for men [10, 11]. There are limited studies on the relation of the insulin indices with CVD risk factors, which indicating controversial findings [12–15]. Nimptsch et al. demonstrated that the II was related to higher triglycerides and lower high-density lipoprotein cholesterol (HDL-C) and showed no association with BS and C-reactive protein (CRP) [15]. In another study, IL indicated no association with triglycerides, HDL-C, and obesity; however, it had a positive association with fasting blood glucose and CRP [14]. Also, IIs are associated with greater odds of obesity and IR [12, 13].

Regarding the well-known association between hyperinsulinemia with the risk of CVD [16], and also the predictive potential of the IIs for determining the insulin response, assessing the relationship between the IIs and development of CVD may give us more practical information about the diet therapy for preventing the risk of CVD. To the best of our knowledge, there is no previous study assessed the relationship between the IIs and the risk of CVD incidence, therefore this study was designed to investigate the association of the dietary II, IL, GI, and GL with the risk of CVD incidence among the Tehranian adults.

## Material and methods

### Study participants

**Fig. 1:**
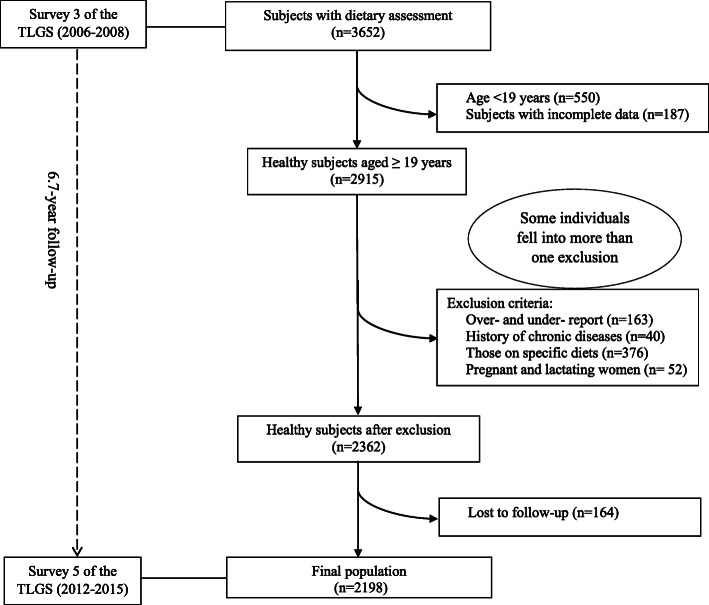
Flow chart of the Tehran Lipid and Glucose Study (TLGS) participants

The present study was fulfilled within the framework of the Tehran Lipid and Glucose Study (TLGS). The TLGS has been performed to assess the risk factors of chronic diseases among Tehranian population, (capital of Iran), which included 15,005 participants aged≥3 years old. This ongoing population-based cohort study initiated in 1999 (baseline phase) and its data are being collected prospectively at 3-year intervals; details of the TLGS have been expressed previously [17]. In the third phase of the TLGS (2006–08), among 12,523 participants, 3652 individuals were randomly considered for nutritional assessment. After excluding the subjects aged< 19 years (*n* = 550) and those with incomplete information (*n* = 187) at the baseline, among 2915 participants aged 19 to 84 years, individuals with a history of myocardial infarction, stroke, and cancer (*n* = 40), those who reported a daily energy intake outside the range of 800–4200 kcal/ day (*n* = 163) or had specific diets (*n* = 376), and pregnant and lactating women (*n* = 52) were excluded from the study; some individuals fell into more than a exclusion category. Finally, 2362 participants were followed-up until March 2015 (Survey 5 of the TLGS:2012–2015), with a mean period of 6.7 years from the baseline examination. After excluding the participants who were missed to follow up (*n* = 164), final analyses were conducted on 2198 adult subjects (Fig. 1).

### Data collection and measurments

Materials and Methods of the TLGS has been described in detail previously [17, 18]. A standard questionnaire was used to determine the information of participants on various socio-demographic variables including sex, age, smoking status, medical history, and drug history. Also, The antropometrics measurments including weight and height, and also blood pressure level of individuals were determined using standard protocols as previously reported [17, 18]. The formula for calculation of body mass index (BMI) is weight in kg divided by height in m^2^. The physical activity level of participants was assessed using Modifiable Activity Questionnaire. The validation of this questionnaire has previously been done for the Iranian population [19]. Furthermore, we took a blood sample at the TLGS research laboratory after 12–14 h of overnight fasting based on the standard protocol to measure fasting blood glucose (FBG) [17, 18].

We used a valid and reliable 168-semi-quantitative food frequency questionnaire (FFQ) to determine the dietary data of patrticipants at the baseline (third phase of the TLGS) [20]. Food intakes of the study population over the previous year on a daily, weekly or monthly basis were assessed by trained and experienced nutrition experts. Using the United States Department of Agriculture (USDA) food composition table (FCT), energy and nutrients content was computed. Local food items that were not available in USDA FCT, was analyzed using The Iranian FCT.

For each carbohydrate-containing food, glycemic index (GI) is defined as the area under the blood glucose response curve over 2 h after eating the food relative to that after consuming the equivalent amount of carbohydrate as glucose or white bread. The GI value of each food item was obtained from the international table of GI and from the publication that lists the GI of Iranian foods [21, 22]. Dietary GI is calculated by multiplying the carbohydrate content of each food item by the number of servings/d and by the GI and then dividing by total daily carbohydrate intake. Dietary GL is also calculated by multiplying the carbohydrate content of each food item by the number of servings/d and by the GI.

We used the published data of three previous studies by Jennie C Brand-Miller at the University of Sydney to measure the insulin indices including II and IL [8, 9, 23]. In the calculation of insulin index (II), postprandial plasma insulin response after consumption of each food compares with a reference food including glucose or white bread. The insulin index value was computed by dividing the area under the insulin response curve for 1000 kJ (239 kcal) of each food by the area under the insulin response curve for 1000 kJ (239 kJ) of the reference food ^8^. We matched the food items of our FFQ with previous food items in which their II was calculated previously by Jennie C Brand-Miller et al. Then, the total dietary insulin load (IL) of the whole diet during the past year for each of the study population by multiplying the insulin index value of each food by the total energy intake contributed by that food and summing values for all food items reported [24]. The dietary II of the whole diet was calculated by dividing the dietary IL by the total energy intake [24].

### Definitions

The detailed information on CVD outcomes ascertainment has been documented previously [25, 26], in which CVD was defined as any coronary heart disease (CHD) events, CVD death (fatal CHD, fatal myocardial infarction (MI), and fatal stroke), or stroke [27]. Coronary heart disease events considered cases of definite MI (diagnostic ECG and biomarkers), probable MI (positive ECG findings, cardiac symptoms or signs, and missing biomarkers or positive ECG findings and equivocal biomarkers), and angiographic confirmed CHD. Stroke in individuals was defined as a new neurological deficit that lasted at least on day. Previous ischemic heart disease and/or cerebrovascular accidents was considered as history of CVD.

We used JNC8 criteria for determining hypertension as systolic blood pressure ≥ 140, or diastolic blood pressure ≥ 90, or using antihypertensive drugs in subjects, aged < 60 years and systolic blood pressure ≥ 150, or diastolic blood pressure ≥ 90 or taking antihypertensive medications in subjects, aged > 60 years [28].

Type 2 diabetes was described as a fasting blood glucose of 126 mg/dl and higher, 2-h blood glucose of 200 mg/dl and higher, or being on anti-diabetic medication [29].

### Statistical analysis

All statistical analyses were performed using the Statistical Package for Social Sciences (Version 15.0; SPSS, Chicago, IL). The histogram charts and Kolmogorov–Smirnov test were used to assess the normality of the variables. Inviduals were categorized based on quartiles of II, IL, GI, and GL. Presentation of the baseline characteristics among subjects was reported according to quartiles of IL and GL; as the mean ± SD or median (interquartile) and percentages for continuous and categorical variables, respectively. To test the trend of qualitative and quantitative variables across quartiles of IL and GL (as the median value in each quartile), Chi-square and linear regression were used, respectively.

Time to event was determined as time to end of follow-up (censored cases) or time to having an event, whichever occurred first. Participants were censored at the time of death because of non-CVD causes, at the time of leaving the district or study follow-up end time of March 2014. We used the Cox proportional hazard models to estimate multivariate-adjusted hazard ratio (HR) and 95% confidence interval (CI) for the CVD incident across quartile of II, IL, GI, and GL and also based on the continues level of these indices (per 1 SD increment). We also adjusted the potential confounders including age, sex, BMI, physical activity, smoking, diabetes, and hypertension, and daily energy intake in Cox regression models. The linear trend of CVD risk in quartiles of insulinemic and glycemic scores was tested by entering the median values of each category as a continuous variable in the different models. The proportional hazards assumption was checked using a log-log plot, and the assumption was satisfied (lines in the plots were parallel). *P*-values < 0.05 were considered to be statistically significant.

## Results

The mean ± SD age and BMI of the participants at baseline (44.9% men) were 38.3 ± 13.4 (19–84) years and 26.6 ± 4.8 Kg/m^2^, respectively. The follow up of participants was started in 2006–2008 and was continued until the end of 2015 (fifth phase: 2012–2015). During the median (IQR) 6.7 (6.1–7.1) year of follow-up, 76 (3.5%) new cases of CVD were ascertained (19–30 years:0 cases, 31–40 years: 1 case, 41–50 years: 17 cases, 51–60 years: 25 cases, 61–70 years: 25 cases, and > 70 years: 8 cases). The mean ± SD of II, IL, GI, and GL of participants were 51.7 ± 6.5, 235.8 ± 90.2, 61.9 ± 7.8, and 202.2 ± 78.1, respectively.

Individuals in the highest quartile of IL score were more likely to be male, physically active, smokers, having hypertension, and had higher dietary intakes of energy, refined grain, carbohydrate, and fiber compared to those with the lowest score. However, participants with higher IL scores had lower age and lower intakes of fruit and vegetable, red and processed meat, nuts and legumes, fat, and protein compared to those with the lowest score. No significant trends were observed in other baseline characteristics across quartiles of IL (Table 1).

**Table 1:** Baseline characteristics of the study population by quartiles of dietary insulin load (*N* = 2198)

Characteristics	Insulin Load				P for trend
	Q1(*n* = 549)	Q2(*n* = 550)	Q3(*n* = 549)	Q4(*n* = 550)	
Insulin load	137 ± 26	196 ± 14	250 ± 17	359 ± 67	< 0.001
Insulin index	48.6 ± 6.4	50.6 ± 6.2	52.6 ± 5.7	55.0 ± 5.9	< 0.001
Age (years)	39.2 ± 13.4	38.3 ± 13.2	38.7 ± 13.2	37.1 ± 13.5	< 0.05
Male (%)	27.1	36.9	52.8	62.5	< 0.001
Body mass index (kg/m2)	26.8 ± 4.8	26.6 ± 4.6	26.6 ± 4.9	26.3 ± 4.9	NS
Physical activity (MET_h/week)	23.6 (9.3–46.1)	23.4 (10.4–47.8)	24.3(10.4–50.7)	24.8 (10.9–55.5)	< 0.001
Current smoker (%)	9.8	10.9	13.3	14.2	< 0.05
Diabetes and pre-diabetes (%)	12.0	10.5	12.9	8.7	NS
Hypertension (%)	8.1	7.7	9.5	12.6	< 0.01
**Dietary intakes**					
Energy (kcal)	1521 ± 396	2019 ± 371	2430 ± 434	3095 ± 529	< 0.001
Fruit and vegetable (serving/d/1000 Kcal)	3.0 ± 1.6	2.8 ± 1.4	2.6 ± 1.3	2.1 ± 1.1	< 0.001
Refined grain (serving/d/1000 Kcal)	2.0 ± 1.1	2.1 ± 1.0	2.3 ± 1.1	2.8 ± 15	< 0.001
Red and processed meat (serving/wk./1000 Kcal)	2.2 (1.3–3.8)	2.3 (1.3–3.7)	2.1 (1.3–3.4)	2.0 (1.1–3.4)	< 0.05
Nut and legume (serving/wk./1000 Kcal)	0.99 (0.59–1.69)	0.96 (0.57–1.60)	0.97 (0.58–1.65)	0.87 (0.48–1.46)	< 0.05
Carbohydrate (% of energy)	54.9 ± 7.4	56.3 ± 6.8	57.9 ± 6.3	60.1 ± 6.7	< 0.001
Fat (% of energy)	33.9 ± 7.4	32.5 ± 6.6	31.1 ± 6.2	28.7 ± 6.3	< 0.001
Protein (% of energy)	13.8 ± 2.6	13.8 ± 2.3	13.4 ± 2.1	13.3 ± 2.2	< 0.001
Fiber (g/1000 Kcal)	14.9 ± 5.6	15.6 ± 5.5	16.6 ± 6.9	18.1 ± 8.2	< 0.001

Data are presented as the mean ± SD or as the median (25–75 IQR) for continuous variables and as percentages for categorical variables

Individuals in the highest quartile of GL were more likely to be male, smoker, and being more physically active, and had higher dietary intakes of energy, refined grain, carbohydrate, and fiber. However, age, intakes of fruit and vegetable, red and processed meat, fat, and protein were all decreased across the quartiles of GL. No significant trend was found across quartiles of GL for BMI, hypertension, diabetes and pre-diabetes, and dietary intakes of nuts and legumes (Table 2).

**Table 2:** Baseline characteristics of the study population by quartiles of dietary glycemic load (*N* = 2198)

Characteristics	Glycemic load				P for trend
	Q1(*n* = 549)	Q2(*n* = 550)	Q3(*n* = 550)	Q4(*n* = 549)	
Glycemic load	117 ± 22	167 ± 12	214 ± 16	309 ± 59	< 0.001
Glycemic index	58.4 ± 7.8	60.8 ± 7.0	62.3 ± 6.8	66.2 ± 7.5	< 0.001
Age (years)	39.2 ± 13.8	38.9 ± 13.1	38.0 ± 13.0	37.1 ± 13.6	< 0.01
Male (%)	28.1	39.8	48.5	63.0	< 0.001
Body mass index (kg/m2)	26.9 ± 4.9	26.6 ± 4.6	26.5 ± 5.0	26.3 ± 4.7	NS
Physical activity (MET_h/week)	23.6 (8.9–45.3)	23.8 (9.1–47.1)	24.5 (11.4–52.1)	24.3 (11.3–55.1)	< 0.01
Current smoker (%)	9.7	10.2	14.5	13.8	< 0.05
Diabetes and pre-diabetes (%)	13.3	10.9	10.4	9.7	NS
Hypertension (%)	9.5	7.3	10.2	10.8	NS
**Dietary intakes**					
Energy (kcal)	1522 ± 386	2012 ± 373	2430 ± 431	3103 ± 524	< 0.001
Fruit and vegetable (serving/d/1000 Kcal)	2.8 ± 1.5	2.7 ± 1.3	2.7 ± 1.4	2.3 ± 1.3	< 0.001
Refined grain (serving/d/1000 Kcal)	1.9 ± 1.0	2.1 ± 1.0	2.3 ± 1.1	3.0 ± 1.5	< 0.001
Red and processed meat (serving/wk./1000 Kcal)	2.3 (1.2–3.8)	2.2 (1.3–3.5)	2.2 (1.4–3.7)	2.0 (1.1–3.4)	< 0.05
Nut and legume (serving/wk./1000 Kcal)	0.97 (0.57–1.63)	0.97 (0.59–1.64)	0.92 (0.53–1.57)	0.95 (0.50–1.57)	NS
Carbohydrate (% of energy)	54.1 ± 7.4	56.3 ± 6.5	58.1 ± 6.5	60.7 ± 6.2	< 0.001
Fat (% of energy)	34.3 ± 7.5	32.4 ± 6.4	30.9 ± 6.3	28.5 ± 6.1	< 0.001
Protein (% of energy)	14.1 ± 2.7	13.8 ± 2.3	13.5 ± 2.2	12.9 ± 1.9	< 0.001
Fiber (g/1000 Kcal)	14.9 ± 5.7	15.8 ± 5.7	16.5 ± 6.6	18.0 ± 8.2	< 0.001

Data are presented as the mean ± SD or as the median (IQR) for continuous variables and as percentages for categorical variables

The association of II, IL, GI, and GL with the incidence of CVD are presented in Table 3. No significant association was found between the II, IL, GI, and GL and the risk of CVD incidence after 6.5 years of follow-up based on the age and sex-adjusted model. However, in the model fully adjusted for potential confounders including age, sex, BMI, physical activity, smoking, diabetes, and hypertension, and daily energy intake, the HR of incidence of CVD in participants who were in the highest quartile of GL was 2.67 (95% CI: (1.00–7.69), P for trend = 0.033) compared to those in the lowest one. In the final adjusted model, other indices showed no significant association with the medium to large effect sizes for increasing the risk of CVD incidence; the HR(95% CI) of CVD incidence were 1.24 (0.65–2.33), 2.08 (0.70–6.19), and 1.55 (0.80–2.99) for II, IL, and GI, respectively in the highest compared to the lowest quartile.

**Table 3:** Relative risk (95% CI) of cardiovascular disease in participants across quartiles of dietary insulin and glycemic scores*

	Quartiles				P for trend
	Q1	Q2	Q3	Q4	
**Insulin Index**					
Median (IQR) score	44.3 (41.4–46.3)	50.0 (49.0–50.9)	53.8 (52.8–55.0)	58.9 (57.3–61.5)	
CVD/Total	15/549	17/550	15/549	29/550	
person-years	3660.2	3605.2	3623.0	3578.8	
Incidence rate (10.000 person year)	40.9 (24.6–67.8)	47.0 (29.2–75.7)	41.3 (24.9–68.5)	80.9 (56.2–116.4)	
Crude model	1.00 (ref)	1.15 (0.57–2.31)	1.01 (0.49–2.07)	**1.98 (1.06–3.69)**	**0.033**
Model 1	1.00 (ref)	1.01 (0.50–2.04)	0.66 (0.32–1.35)	1.10 (0.58–2.08)	0.840
Model 2	1.00 (ref)	1.16 (0.57–2.36)	0.69 (0.33–1.45)	1.24 (0.65–2.33)	0.638
**Insulin Load**					
Median (IQR) score	142.6 (118.8–159.0)	196.0 (183.2–209.4)	248.5 (235.2–264.8)	340.6 (307.4–393.3)	
CVD/Total	15/549	18/550	22/549	21/550	
person-years	3643.2	3643.5	3630.9	3550.1	
Incidence rate (10.000 person year)	41.1 (24.7–68.1)	49.3 (31.0–78.2)	60.4 (39.8–91.8)	59.0 (38.5–90.5)	
Crude model	1.00 (ref)	1.19 (0.60–2.37)	1.47 (0.76–2.83)	1.41 (0.73–2.75)	0.279
Model 1	1.00 (ref)	1.26 (0.63–2.50)	1.20 (0.61–2.35)	1.38 (0.71–2.71)	0.386
Model 2	1.00 (ref)	1.52 (0.71–3.27)	1.69 (0.71–3.77)	2.08 (0.70–6.19)	0.238
**Glycemic Index**					
Median (IQR) score	53.0 (49.6–55.1)	59.9 (58.7–61.0)	64.4 (63.3–65.6)	70.6 (68.6–73.3)	
CVD/Total	18/550	24/549	15/549	19/550	
person-years	3587.6	3603.9	3644.7	3629.6	
Incidence rate (10.000 person year)	50.0 (31.5–79.5)	66.4 (44.5–99.2)	41.0 (24.7–68.1)	52.2 (33.3–81.9)	
Crude model	1.00 (ref)	1.34 (0.72–2.47)	0.82 (0.41–1.64)	1.05 (0.55–2.00)	0.826
Model 1	1.00 (ref)	1.30 (0.70–2.41)	0.87 (0.43–1.73)	1.27 (0.66–2.43)	0.703
Model 2	1.00 (ref)	1.32 (0.71–2.46)	0.97 (0.48–1.97)	1.55 (0.80–2.99)	0.305
**Glycemic Load**					
Median (IQR) score	120.6 (104.2–134.8)	167.5 (157.5–178.5)	213.5 (200.9–227.4)	292.1 (265.3–333.1)	
CVD/Total	18/549	16/550	17/550	25/549	
person-years	3606.5	3677.1	3613.3	3570.9	
Incidence rate (10.000 person year)	49.8 (31.3–79.0)	43.4 (26.6–70.9)	46.9 (29.2–75.5)	69.9 (47.2–103.4)	
Crude model	1.00 (ref)	0.87 (0.44–1.71)	0.94 (0.48–1.83)	1.39 (0.76–2.56)	0.192
Model 1	1.00 (ref)	0.88 (0.44–1.73)	0.97 (0.50–1.91)	1.42 (0.76–2.65)	0.170
Model 2	1.00 (ref)	1.15 (0.54–2.44)	1.53 (0.66–3.50)	**2.77 (1.00–7.69)**	**0.033**

*Cox proportional hazard regression models were used to estimate relative risks (RR) and 95% confidence interval (CI)

Model 1: Adjusted for age and sex

Model 2: Additionally adjusted for body mass index, physical activity, smoking (yes or no), daily energy intake, diabetes, and hypertension

Table 4 shows the association between each SD increasing the insulin and glycemic scores and the risk of CVD incident among all participants. In the crude model, per one SD increasing the II score, the HR (95% CI) of CVD was 1.29 (1.02–1.62), *P*-value = 0.029. In the final model, after adjusting for potential confounders, each SD increase in the GL score was associated with a higher risk of CVD [(RR:1.46; CI:1.00–2.16), *P*-value = 0.047]. However higher score of II, IL, and GI showed no significant association with the incidence of CVD.

**Table 4:** Relative risk (95% CI) of cardiovascular disease in participants per one SD increase in the dietary insulin and glycemic scores*

Indices	Relative Risk (95% CI)	***P***-value
**Insulin Index**		
One SD of score	6.56	
Crude model	**1.29 (1.02–1.62)**	**0.029**
Model 1	1.00 (0.80–1.26)	0.998
Model 2	1.03 (0.82–1.30)	0.739
**Insulin Load**		
One SD of score	90.20	
Crude model	1.10 (0.88–1.36)	0.393
Model 1	1.07 (0.85–1.35)	0.546
Model 2	1.11 (0.74–1.65)	0.597
**Glycemic Index**		
One SD of score	7.84	
Crude model	1.02 (0.81–1.28)	0.819
Model 1	1.09 (0.86–1.37)	0.424
Model 2	1.16 (0.92–1.47)	0.185
**Glycemic Load**		
One SD of score	78.18	
Crude model	1.11 (0.90–1.38)	0.279
Model 1	1.15 (0.92–1.43)	0.186
Model 2	**1.46 (1.00–2.16)**	**0.047**

*Cox proportional hazard regression models were used to estimate relative risks (RR) and 95% confidence interval (CI)

Model 1: Adjusted for age and sex

Model 2: Additionally adjusted for body mass index, physical activity, smoking (yes or no), daily energy intake, diabetes, and hypertension

In a subsample of the participants with dysglycemia (*n* = 243), it was observed that those in the highest quartile of II, IL, GI, and GL had a higher risk of CVD with HR(95% CI) of (1.20 (0.43–3.32), P for trend = 0.610), (5.59 (0.96–32.55), P for trend = 0.046), (3.24 (0.96–11.00), P for trend = 0.097), and (5.66 (1.08–29.61), P for trend = 0.029), respectively compared to those in the lowest quartile.

## Discussion

The current study showed that the highest score of dietary GL was significantly associated with an increased risk of CVD, independently of potential confounding factors including age, sex, BMI, physical activity, smoking, diabetes, hypertension, and daily energy intake in the Tehranian adults. However, there was no significant association between the dietary GI, II, and IL score and the risk of CVD in the adults.

In the last decade, meta-analyses and review reports have summarized the findings on the association of GI and GI with the risk of CVD events [10, 11, 30]; our findings are comparable with the results of these previously published studies conducted on the adult population reporting that high dietary GL and GI are associated with the increased risk of CVD events, specifically for women. Three review studies have suggested that gender may significantly modify the association of dietary GL and GI with the risk of CVD outcomes [10, 11, 30]. Xiang-yu et al. revealed that high dietary GL and GI are related to a 23and 13% increase in the risk of CVD events, respectively. This estimated risk of GL and GI was lower for men than women. This dose-response meta-analysis has found an increased risk of CVD events by 18% per 50 unit increment of GL in the diet [10]. Also, based on the findings of two previously published review studies, a diet with high GL and GI significantly increased the risk of CVD events in women but not men [11, 30]. Knopp et al. reported that the increase in the triacylglycerol and the decrease in the high-density lipoprotein cholesterol concentration in response to a high glycemic diet are greater in the women than the men, which led to the differences in the results of the men and women [31].

Iranians consume more than 55% of energy from the carbohydrate, which is higher than the amounts consumed in most western countries [32]. Our population has high carbohydrate intakes from plant sources, such as refined grain, rice, and potato [33, 34]. Therefore, in our study, white rice and bread especially white bread were the major contributors to dietary GL and GI; as these food items have a high content of carbohydrates and a low amount of fiber, possibly contributing to the development of cardio-metabolic risk factors. However, higher adherence to the low GL diet characterized by high consumption of whole-grain bread, fruits, vegetables, and legumes with high fiber content, if combined with lower consumption of white bread and rice could have a protective role in decreasing the CVD risk [35, 36].

Although the insulin response is almost induced by the carbohydrates, other nutrients, such as dietary protein and fats can affect insulin secretion. Protein-rich foods or some fats can induce the insulin response; also dietary protein augments the insulin response after consuming carbohydrate-containing foods [8]. Recently, previous studies have reported that the dietary II and IL can be more applicable to assess the effect of insulin exposure on the development of the non-communicable chronic diseases than other factors, such as GI, GL, or total carbohydrate intake; because II is directly based on the insulin response and this index could directly quantify the insulin secretion in response to the carbohydrate and protein-rich foods [37]. Despite the lack of longitudinal study on the association of the dietary II and IL with CVD risk, there are several studies reported the controversial findings on the association of dietary II or IL with cardiovascular risk factors, including hyperglycemia, insulin resistance, obesity, high level of CRP, and dyslipidemia [12–15]. Nimptsch et al. showed that dietary II is directly associated with the low HDL-C and high triglycerides levels in obese individuals; however, there are no association between the dietary II and IL and low-density lipoprotein-cholesterol, CRP, and biomarkers of glycemic control [15]. In a cohort study conducted within the framework of TLGS, dietary II and IL were mentioned as the independent dietary risk factors for the risk of insulin resistance [13]. Also, a cross-sectional study has reported that the high II diet is associated with higher odds of obesity among the women, but not men [12]. Furthermore, Mozaffari et al. indicated that the adherence to a high IL diet is positively related to the serum fasting blood glucose and hs-CRP levels. However, no association was reported between the high dietary IL and BMI or lipid profiles [14].

This study is the first study assessed the association of dietary II and IL with the incidence of CVD events and our results showed no significant association between scores of insulin indices and the risk of CVD. Our failure to find a significant association between dietary II and IL and the risk of CVD may be due to some reasons. First, due to the lack of dietary insulin data, some food items of FFQ, such as some fruits or vegetables were not included in the final dietary II and IL scoring, which could be effective on this association. Second, in the current study, individuals in the highest quartile of dietary IL had higher intakes of fiber and lower intakes of fat compared to those in the lowest one; in addition to influencing on the calculated dietary IL score, these nutritional factors can also have a modifying effect on the association between the insulin indices and development of CVD, independently in the form of a healthy diet. Third, the insulin index values of food items were derived from reports conducted on the young lean students whose insulin responses can be relatively different from our adult and elderly participants [8]. Fourth, the incidence rate of CVD events was significantly higher in the participants in the fourth quartile of the IL than those in the lowest quartile (Q4: 59.0 per 10,000 person-years vs. Q1: 41.1 per 10,000 person-years). This significant difference in the incidence rate of CVD was also observed in the participants based on quartiles of GL (Q4: 69.9 per 10,000 person-years vs. Q1: 49.8 per 10,000 person-years). However, the low power of the study due to the low sample size and a limited number of CVD cases in each quartile of IL led to provide non-significant findings in our study.

A subgroup analysis in this prospective cohort study on 243 individuals with dysglycemia revealed that the dietary pattern with high GL and IL can be related strongly with increased risk of CVD in high-risk participants. It has been previously reported that most individuals with dysglycemia have both hyperinsulinemia and insulin resistance and they are prone to CVD outcomes, such as coronary heart disease and stroke. Therefore, a dietary pattern with high GL and IL is likely to be associated with an increased risk of insulin resistance, atherogenesis, and subsequently an increased risk of CVD [38].

Some reports have suggested supporting mechanisms and pathways, which can explain a beneficial association between these indices and the risk of CVD incidence. The beneficial effect of low GL or GI diets on reducing cardio-metabolic risk factors, including total cholesterol, low-density lipoprotein-cholesterol, triglycerides, BMI, and plasminogen activator inhibitor-1 concentrations have also been reported previously [39, 40]. Also, the high dietary GI is associated with higher plasma levels of tumor necrosis factor-a, interleukin 6, and C-reactive protein [40, 41]. Furthermore, blood glucose concentration increases, and stimulates insulin release after consuming high-GI foods with the same amount of carbohydrate,. The chronically increased insulin demand may eventually lead to the destruction of pancreatic β cells, and consequently resulting in impaired glucose tolerance. Also, high-GI diets can directly increase insulin resistance through their effect on glycemia and free fatty acids [42]. Some reports have suggested the potential mechanisms through which dietary II and IL might influence the CVD risk factors. High II of diet may increase the risk of obesity by stimulating more insulin secretion, which can reduce fat oxidation and increase carbohydrate oxidation, causing an increase in fat storage [43]. Also, high dietary IL and II can be linked to β-cell dysfunction and increased insulin resistance through influencing insulin secretion [13].

The current study is subject to some limitations; the Iranian food composition table was incomplete and the USDA nutrient databank was mostly used for the dietary analyses. Also, it will be difficult to generalize our findings to other societies because of the differences in the food culture and food intake values of the study participants. Furthermore, despite adjusting for a wide variety of variables in our analysis, the confounding effect of some unknown or unmeasured residual confounding may have occurred. Despite these limitations, to date, this is the first study that assessed the association of dietary II and IL with the incidence of CVD conducted in the Middle East and North Africa region. The prospective setting, long duration of follow-up, and the use of valid and reliable food-frequency and physical activity questionnaires were other strengths of the current study.

## Conclusions

In conclusion, the findings of the current population-based cohort study revealed that a dietary pattern with higher GL is associated with the increased risk of CVD; however, no association was found between the dietary GI, II, and IL and the risk of CVD. Further observational studies with long-term follow up are required to address the role of a diet with high II and IL in the development of CVD outcomes and its potential mechanisms.

## Data Availability

The datasets analyzed in the current study are available from the corresponding author on reasonable request.

## Abbreviations

BMI: Body mass index
CI: Confidence interval
CRP: C-reactive protein
CVD: Cardiovascular disease
FCT: Food composition table
FFQ: Food frequency questionnaire
GI: Glycemic index
GL: Glycemic load
HRs: Hazard ratios
II: Insulin index
IIs: Insulin indices
IL: Insulin load
IR: Insulin resistance
MET-h/wk.: Metabolic equivalent hours per week
MI: Myocardial infarction
TLGS: Tehran Lipid and Glucose Study
USDA: United States Department of Agriculture
